# Zinc Oxide Nanoparticles Induce an Adverse Effect on Blood Glucose Levels Depending On the Dose and Route of Administration in Healthy and Diabetic Rats

**DOI:** 10.3390/nano10102005

**Published:** 2020-10-12

**Authors:** Adolfo Virgen-Ortiz, Alejandro Apolinar-Iribe, Irene Díaz-Reval, Hortensia Parra-Delgado, Saraí Limón-Miranda, Enrique Alejandro Sánchez-Pastor, Luis Castro-Sánchez, Santos Jesús Castillo, Adan Dagnino-Acosta, Edgar Bonales-Alatorre, Alejandrina Rodríguez-Hernández

**Affiliations:** 1 Centro Universitario de Investigaciones Biomédicas, Universidad de Colima, Colima C.P. 28045, Mexico; idiazre@ucol.mx (I.D.-R.); espastor@ucol.mx (E.A.S.-P.); ebonales0@ucol.mx (E.B.-A.); 2 Departamento de Física, Universidad de Sonora, A.P. 1626, Hermosillo, Sonora C.P. 83000, Mexico; apolinar@ciencias.uson.mx; 3 Facultad de Ciencias Químicas, Universidad de Colima, Coquimatlán, Colima C.P. 28400, Mexico; hparra@ucol.mx; 4 Departamento de Ciencias Químico Biológicas y Agropecuarias, URS, Universidad de Sonora, Navojoa, Sonora C.P. 85880, Mexico; sarai.limon@unison.mx; 5 Centro Universitario de Investigaciones Biomédicas, CONACYT-Universidad de Colima, Universidad de Colima, Colima C.P. 28045, Mexico; luis_castro@ucol.mx (L.C.-S.); dagninoa@ucol.mx (A.D.-A.); 6 Departamento de Investigación en Física, A.P. 5-088, Hermosillo, Sonora C.P. 83000, Mexico; santos.castillo@unison.mx; 7 Facultad de Medicina, Universidad de Colima, Colima C.P. 28040, Mexico; arodrig@ucol.mx

**Keywords:** zinc oxide nanoparticles, diabetes, hyperglycemic response, zinc, nanomedicine, nanoparticle toxicology

## Abstract

Different studies in experimental diabetes models suggest that zinc oxide nanoparticles (ZnONPs) are useful as antidiabetic agents. However, this evidence was performed and measured in long-term treatments and with repeated doses of ZnONPs. This work aimed to evaluate the ZnONPs acute effects on glycemia during the next six h after an oral or intraperitoneal administration of the treatment in healthy and diabetic rats. In this study, the streptozotocin-nicotinamide intraperitoneal administration in male Wistar rats were used as a diabetes model. 10 mg/kg ZnONPs did not modify the baseline glucose in any group. Nevertheless, the ZnONPs short-term administration (100 mg/kg) induced a hyperglycemic response in a dose and route-dependent administration in healthy (130 ± 2 and 165 ± 10 mg/dL with oral and intraperitoneal, respectively) and diabetic rats (155 ± 2 and 240 ± 20 mg/dL with oral, and intraperitoneal, respectively). The diabetic rats were 1.5 fold more sensitive to ZnONPs effect by the intraperitoneal route. In conclusion, this study provides new information about the acute response of ZnONPs on fasting glycemia in diabetic and healthy rat models; these data are essential for possible future clinical approaches.

## 1. Introduction

Worldwide, a decade ago, more than 30,000 t of ZnONPs were produced annually [1], surely today it has increased significantly due to its wide industrial application in cosmetics, sunscreens, coatings, paints and antimicrobials. ZnONPs have shown catalytic, electrical, photochemical, anticorrosive, photovoltaic, antifungal, antibacterial, and antiviral activity [2]. In the biomedical field, ZnONPs have been used for development of biosensors for a wide variety of molecules of interest, to improve diagnosis through imaging, controlled drug release, gene delivery and as therapeutic agents [3,4,5,6]. There is promising scientific evidence for the treatment of diseases with a high worldwide prevalence where several studies evaluated the anticancer, antidiabetic and antimicrobial activity of ZnONPs [7,8,9]. The whole potential of the application of these nanoparticles for the benefit of humans, demands studies and a detailed understanding of all their possible toxic or adverse effects on human health and the environment. In the literature, some toxic effects of ZnONPs have been described, these vary according to factors, such as their physicochemical characteristics, the concentrations, doses, exposure time and the route of administration used in the experiments. In general, it is suggested that the toxic effects produced by ZnONPs in different tissues or cell lines are mediated by increased oxidative stress and inflammation [7,10].

On the other hand, in the balance of benefits versus toxicology of metal nanoparticles for the treatment of mellitus diabetes, different studies have described that metal nanoparticles (silver, gold, cooper, selenium, magnesium, cerium oxide, titanium dioxide [11,12,13,14,15,16,17,18] and zinc oxide possess antihyperglycemic activity in diabetic rats after daily treatment for different periods [8,19,20,21,22,23].

In particular, although ZnONPs have been reported to have antihyperglycemic activity, studies in this regard are scarce. For this reason, more detailed research is required to determine their importance as therapeutic agents in chronic treatments. A recent study, demonstrated that an oral administration of 1–10 mg/kg during 4 weeks reduced hyperglycemia in type 1 diabetes (D1) and type 2 diabetes (D2), but the insulin level was not affected in D1. In contrast, insulin levels only increased at a dose of 10 mg/kg in D2, explaining the improved glucose tolerance in this model [19]. A similar effect was observed after a seven-weeks treatment [22]. However, other related studies using the identical administration route during four or eight weeks showed an increase in insulin levels associated with the antihyperglycemic effects in D1 [8,23]. The critical evidence supporting that oral nanoparticles administration in a dose range of 1–10 mg/kg/day for several weeks has antidiabetic activity; nonetheless, the immediate effect ZnONPs post-administration on basal glycemia has not been studied and its evaluation is essential to detect a possible risk in the diabetic patient since both an increase or a drastic decrease in glucose levels compromises their health., e.g., the acute hypoglycemia and hyperglycemia have been reported to induce atherothrombotic effects in non-diabetic and diabetic individuals, and these alterations have been associated with an increase in morbidity and mortality caused by cardiovascular failure [24,25,26]. Furthermore, an acute drastic imbalance in blood glucose levels in a diabetic patient can induce a potentially fatal diabetic coma [27].

The lack of this information limits the integral control of alterations suffered by diabetic patient. The goal of this research was to evaluate the acute effects on glycemia of oral and intraperitoneal administration of ZnONPs in healthy and diabetic rats.

## 2. Materials and Methods 

### 2.1. Material

Zinc oxide nanoparticles dispersion (Cat. No. 721077, density 1.7 g/mL), nicotinamide (Cat. No. N3376, purity ≥ 98% HPLC), and streptozotocin (Cat. S0130, purity ≥ 98% HPLC) were obtained from Sigma-Aldrich Co. (St Louis, MO, USA); a sterile saline solution of sodium chloride (0.9%) was acquired from PISA Pharmaceutical Co. (Jalisco, Mexico). 

It has been reported that ZnONPs in aqueous media is unstable depending on the concentration, pH and ionic strength of the medium [28]. However, it has also been described that sonication produces a stable suspension useful for biological assays [29]. Therefore, in our study for in vivo tests, a suspension of ZnONPs was freshly prepared at a concentration of 10 mg/mL as follows: The ZnONPs were deposited in a sterile saline solution and subsequently sonicated for 10 min (50% pulse amplitude with resting times of 30 seconds between pulses, 130 Watts, 20 KHz Ultrasonic Processor (Cole-Palmer Instruments, Vernon Hills, IL, USA).

### 2.2. ZnONPs Characterization

The shape and size of the nanoparticles were determined using scanning transmission electron microscopy (STEM, JEOL, JSM-7800F, Pleasanton, CA, USA) in an aliquot of ZnONPs suspension. The hydrodynamic diameter was measured in a previously sonicated suspension of ZnONPs (dissolved in 0.9% NaCl), the measurement based on dynamic light scattering (DLS) was performed using a ZetaPlus size analyzer (Brookhaven Instruments Co., Holtsville, NY, USA).

### 2.3. Animals

For this study, intact three-month-old male Wistar rats (n = 96) were used, this particular age was selected to reduce the streptozotocin sensitivity, since this drug induces experimental diabetes with known higher sensitivity in very young rats [30].

The rats were maintained in individual cages with water and food *ad libitum* (Rodent Laboratory Chow 5001, PMI Nutrition International LLC). They were kept in a room with light-dark cycles (12 h/12 h) and room temperature control (25 °C). During a week prior to the start of the experiment, all rats were manipulated for their adaptation and to eliminate manipulation stress at the time of performing glucose measurements in vivo. All studies were conducted in accordance with the Guide for the Care and Use of Laboratory Animals published by the US National Institute of Health (NIH) and approved by the Bioethics Committee of the University of Colima (Approval number 2018-15).

### 2.4. Experimental Design and Diabetes Induction

The rats were divided into 2 groups: Diabetic rats (n = 48) and non-diabetic rats (n = 48). Experimental diabetes in rats was induced by an intraperitoneal sequential treatment with streptozotocin and nicotinamide. First, streptozotocin was dissolved in citrate buffer pH = 4.5 and then administrated (65 mg/kg body weight). After fifteen min, nicotinamide dissolved in 0.9% saline solution was injected (230 mg/kg body weight). This model induces partial cytotoxicity on pancreatic β-cells producing moderate hyperglycemia without body weight loss or drastic decreases of plasma insulin levels [31]. After seven days, the glycemia was measured in blood samples collected from rat tail using an Accu-chek^®^ Performa blood glucose system (Roche Diagnostics, Mannnheim, Germany); rats with fasting glucose of 126 mg/dL were included in the diabetes group (World Health Organization).

### 2.5. Evaluation of Zinc Oxide Nanoparticles on Fasting Glycemia Values

All rats were fasted for 8 h (07:00 am–03:00 pm) before evaluation. Both groups, diabetic and non-diabetic rats, were subdivided (n = 8 by subgroup) for the test of two doses of ZnONPs, 10 and 100 mg/kg body weight by two administration routes, oral or intraperitoneal. Before each administration, the ZnONPs dispersion was previously vortexed for 30 seconds to maintain its homogeneity.

Glycemia was evaluated at time 0 and 15, 30, 60, 90, 120, 240, and 360 min ZnONPs post-administration using an Accu-chek^®^ Performa blood glucose monitor (Roche Diagnostics, Mannnheim, Germany). The blood sample was obtained from the distal part of pre-cleaned rat tail using an alcohol swab; immediately after, a small cut was made with scissors and the blood obtained is deposited on the test strip and placed on the digital glucometer. The clot was removed for future fresh blood collection to perform the glucose measurement. This procedure is repeated with each rat. 

### 2.6. Statistics

All data is expressed as mean ± standard error. Experimental results were analyzed using a one-way ANOVA with post hoc test (Bonferroni) for statistical differences among groups. Differences with *p* < 0.05 were considered significant. 

## 3. Results and Discussion

**Figure 1:**
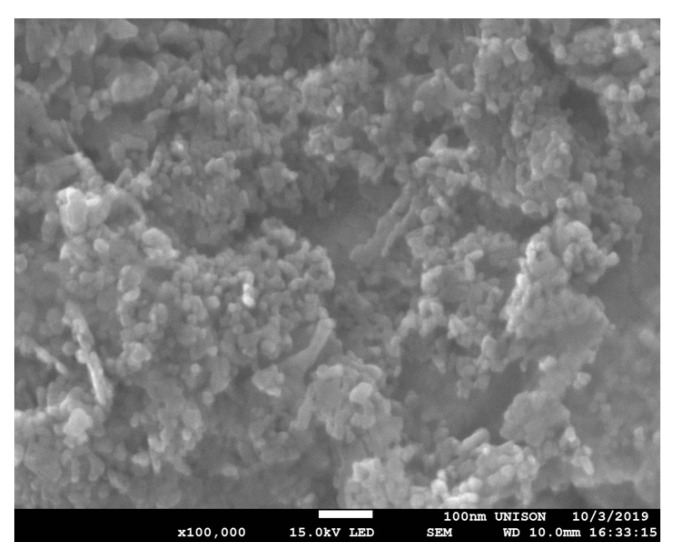
STEM imagen of ZnONPs shows a spherical shape. The scale bar represent a 100 nm length.

The images obtained by STEM demonstrated that the ZnONPs have a spherical shape, and the size analysis performed with ImageJ software determined that they have an average diameter of 17 ± 3.6 nm (Figure 1). DLS analysis revealed that ZnONPs dissolved in saline solution have an average hydrodynamic diameter of 1455 nm and polydispersity index of 0.48.

**Figure 2:**
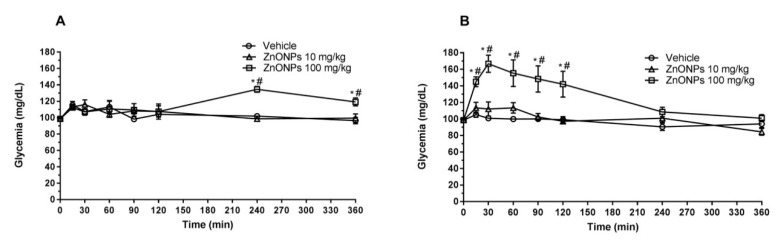
Short-term effects on glycemia of oral or intraperitoneal administration of ZnONPs in fasted healthy rats. (**A**) Oral administration (p.o). (**B**) Intraperitoneal route (i.p). Vehicle (Sterile 0.9% sodium chloride solution). * significant in comparison with vehicle (*p* < 0.05, n = 8 by group), # significant difference in comparison with group treated (10 mg/kg).

**Figure 3:**
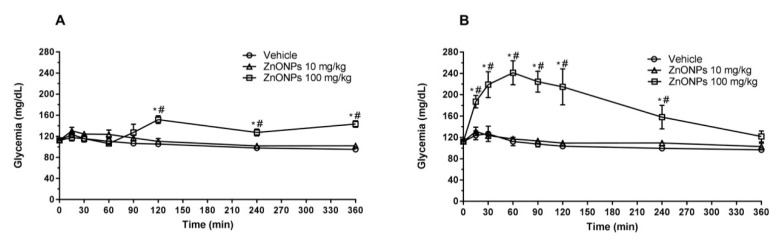
Short-term effects on glycemia of oral or intraperitoneal administration of ZnONPs in fasted diabetic rats. (**A**) Oral administration (p.o). (**B**) Intraperitoneal route (i.p). Vehicle (Sterile 0.9% sodium chloride solution). * significant in comparison to vehicle (*p* < 0.05, n = 8 by group), # significant in comparison with group treated with 10 mg/kg.

ZnONPs dispersion intraperitoneally administered with a 100 mg/kg single dose generated a significant increase in glycemia, compared with the control group treated with vehicle (*p* < 0.05), reaching a maximum peak 30 min after the administration in healthy rats (Figure 2B) and 60 min after in diabetic rats (Figure 3B). The increased levels of blood glucose returned basal levels 6 h post-administration and reached higher levels in diabetic rats when compared with the healthy control group (*p* < 0.05). In contrast, the low dosage tested in this study of 10 mg/kg ZnONPs intraperitoneally administered with same conditions described above generated undistinguishable effects at least in the time range monitored of 6 h (*p* > 0.05) in healthy and diabetic rats.

Oral administration of ZnONPs (100 mg/kg) significantly increased the glucose levels with a lag of 2 h after the administration. This increase was sustained for additional 4 h, in both healthy (Figure 2A) and diabetic rats ( Figure 3A). 

ZnONPs administered intraperitoneally (100 mg/kg) to diabetic rats showed an hyperglycemic induction significantly higher than oral supplementacion using the same dose. However, six hours post-administration, the i.p group showed a normal glycemia with no significant differences in comparison with the oral administration group (Figure 2B and Figure 3B).

As mentioned in the background, it is widely described that ZnONPs have antidiabetic activity when administered for long periods of time. However, their immediate post-administration effects were not studied before. We tested whether they had a hypoglycemic effect with two different doses or affects related to the route of administration. To our surprise, the results showed that ZnONPs do not produce hypoglycemic effects in the short-term, and on the contrary, induce a hyperglycemic response depending on the dose, route of administration and health status (diabetes). To our knowledge, this is the first report demonstrating the ability of ZnONPs to generate short-term hyperglycemic response through a currently unknown mechanism. 

The anti-diabetic activity of ZnONPs in the long term is proposed to be carried out as a result of the stimulation of several mechanisms, among them are the suggestion of an increase of serum insulin levels, glucokinase activity, and increased of insulin, insulin receptor A, GLUT-2 (Glucose transporter 2), and glucokinase mRNA (messenger Ribonucleic Acid) expression [8], reduction in oxidative stress [19,22], less damage to the pancreatic structure [32,33] and microRNA-103 and microRNA-143 decreased expression [23]. In vitro experiments revealed that ZnONPs attenuate the hyperglycemia through a mechanism that involves α-amylase inhibition and α-glucosidase activity [34]. Moreover, in vitro experiments showed that ZnONPs induce GLUT-4 (Glucose transporter 4) translocation and increase β-cell proliferation [35].

In contrast, the lack of knowledge about the mechanism involved in the short-term hyperglycemic response induced by ZnONPs generates new research questions for future work. It is widely known that the liver is the main organ generator of free glucose and is an essential target in antidiabetic therapies [36]. The hyperglycemic effect reported in the present work could be the result of a direct action of a high concentration of zinc ions on the hepatic metabolism; in hepatocytes, zinc at high levels induces an increase of glucose production through glycogenolysis [37]. The zinc supplementation in rats produces a hyperglycemic response, an increase of glucagon, a decrease of insulin, depletion of hepatic glycogen, and hyperglycemia attenuation when the adrenal glands were previously removed [38]. Despite these studies, the action mechanism evidence of a hyperglycemic response by zinc supplementation in the short-term is insufficient, and our results with ZnONPs increase the interest for future research with clinical approaches as antidiabetic agents. 

In the short-term, ZnONPs supplementation could induce a hyperglycemic response by inhibition of insulin secretion. A report in β-cell islets showed that zinc inhibits insulin secretion concentration-dependent [39] and a recent study exhibited that zinc is a critical factor for synthesis and insulin secretion in β-cell [40]; ZnONPs could be dysregulating the insulin secretion pathway at any step in pancreatic β-cell. However, new studies are required to test the hypothesis here stated. 

The differences observed in the temporal courses of hyperglycemic responses in healthy and diabetics can also be a result of a zinc homeostasis alterations, where capture and release are finely modulated to maintain a steady zinc concentration in the cells. The zinc transport is mainly performed by proteins controlling the influx (ZIP) and efflux (ZnT) [41]. There is evidence in the literature that shows differences in the gene expression of zinc transporters in healthy and diabetic rats. In diabetic rats a decrease in the expression of ZIP1 and ZIP4 is observed, which is associated with an over-expression of ZnT1, ZnT2, ZnT4, ZnT5, and ZnT7, which reduces the zinc bioavailability [42].

Pharmacokinetic data of the ZnONPs allows to better understand their effects and toxicity when are administered by different routes. In the experiments carried out in the present work, the hyperglycemic response induced by ZnONPs was lower when they were administered orally, compared to intraperitoneally route, the magnitude of this effect may be due to the fact that the absorption of ZnONPs is low through the gastrointestinal tract (6.5–32.5%), as has been demonstrated in previous studies [43]. On the other hand, it has been reported that clearance is higher when nanoparticles are orally administered in comparison with the intraperitoneal route. In fact, in oral administrations the maximum peak of zinc concentration in blood is reached six h after administration with a subsequent decrease to basal levels. Conversely, using the intraperitoneal route, the concentration is kept high 74 h after the administration, facilitating a greater biodistribution and accumulation of zinc in liver, spleen, lung, kidney and heart [44]. Furthermore, it has been shown that oral administration of ZnONPs generate a rapid clearance by defecation [43,44]. These differences in absorption and clearance could explain the reasons that ZnONPs intraperitoneally injected were more effective in our study. Although, further investigations are required. 

Another interesting explanation for the hyperglycemic phenomenon observed in our experiments would be to study in mammals whether the ZnONPs induce post-administration an imbalance at the systemic level of the hormones that maintain glucose homeostasis, a decrease in insulin coupled with an increase in glucagon and cortisol could induce a hyperglycemic response, in this context there is scientific evidence in other species that shows that ZnONPs decrease the amount of insulin and increase glucagon and cortisol [45]. 

It is important to keep in mind in pharmacological research the dose ranges with no adverse or toxic effects, for this, the toxicity index called NOAEL (No-Observed-Adverse-Effect-Level) is used as an approximation. The NOAEL index for ZnONPs in rats is around 125 mg/kg [46]. In our study, we evaluated two doses below this range, 10 and 100 mg/kg because several reports argue that higher doses (250–2000 mg/kg) generate histological alterations in the liver [43,47]. Such alterations are directly related to the increase in ALT (Alanine aminotransferase) and AST (Aspartate aminotransferase) levels in studies carried out in mice [44,48]. Zinc is significantly absorbed in kidneys, which show damage in histopathological studies. However, creatinine and urea are not altered [48]. Is also known that body weight of rats administered with nanoparticles did not change with doses of 50 to 2000 mg/kg [43]. Bioaccumulation of zinc is also observed in pancreas, liver, and fatty tissue [49]. ZnONPs at doses of 10 mg/kg show no pathological or structural abnormalities in organs such as the liver, kidney, and pancreas [49]. Although, we tested with doses below the NOEL index, the results show that there are doses and routes of administration that can put the life of the diabetic at risk, the study also provides evidence that more detailed toxicology studies are required before thinking about its clinical use.

Finally, another important factor that may be responsible for the difference in the effects observed in this study between oral and intraperitoneal route of administration are the characteristics of the environment of ZnONPs. It has been reported that ZnONPs in gastric fluid increase their hydrodynamic size, and negative surface charge decreases, in contrast when they are placed in plasma ZnONPs decrease in size and increase negative surface charge [50].

In general, the results obtained in this research suggest that any treatment based on ZnONPs in diabetic patients should be taken with caution until an integral evaluation of the risk for adverse effects in future research is performed, including the risk of diabetic coma and compromise of life.

## 4. Conclusions

In the short-term, ZnONPs induce a hyperglycemic response in healthy and diabetic rats; the magnitude of the effect was dose and administration route-dependent. Besides, the hyperglycemic response was higher in diabetic animals. This study provides new information about of acute effects of ZnONPs on the circulating blood glucose levels that could limit its therapeutic application in diabetic patients. Nevertheless, future investigation is required to elucidate the mechanism of action of this compound.

To better understand the acute hyperglycemic effect induced by the ZnONPs, it would be essential to measure in a future study insulin, glucagon and cortisol levels in vivo after the administration of nanoparticles (0–6 h) and explore the effects on the liver metabolism. These experiments will allow us to know whether the ZnONPs act by an imbalance in hormones that regulate blood glucose or increasing hepatic glycogenolysis.
